# Relationship between reduced serum ceruloplasmin levels and executive dysfunction in hospitalized schizophrenia patients

**DOI:** 10.3389/fpsyt.2025.1618147

**Published:** 2025-08-14

**Authors:** Dan Kuang, Hongkai Wei, Wei Qin

**Affiliations:** ^1^ Severe Psychiatric Ward, Shaoxing Seventh People's Hospital (Affiliated Mental Health Center, Medical College of Shaoxing University), Shaoxing, Zhejiang, China; ^2^ Psychiatry Department, Daizhuang Hospital in Shandong Province, Jining, Shandong, China; ^3^ Psychiatry Department, Shandong Mental Health Center Affiliated to Shandong University, Jinan, Shandong, China

**Keywords:** schizophrenia, executive dysfunction, serum ceruloplasmin, Tower of London test, cognitive biomarkers

## Abstract

**Objective:**

Executive dysfunction is a widespread and complex manifestation in schizophrenia, significantly impairing patients’ cognitive and functional outcomes. Despite extensive research, specific biomarkers associated with this dysfunction remain unidentified. Serum ceruloplasmin (Cp), a copper-binding protein involved in iron metabolism and oxidative stress regulation, has recently been implicated in neurological conditions. This study aims to investigate the relationship between reduced serum Cp levels and executive dysfunction in hospitalized schizophrenia patients, providing insights into potential biomarkers and therapeutic targets.

**Methods:**

A total of 95 schizophrenia inpatients treated at Shaoxing Seventh People’s Hospital from January 2023 to December 2024 were enrolled. Patients were divided into two subgroups based on serum Cp concentrations: 48 patients with reduced Cp levels (SC1 group, Cp < 200 mg/L) and 47 patients with normal Cp levels (SC2 group, 200-600 mg/L). Additionally, 42 age- and gender-matched healthy individuals served as the control group. Blood samples were collected for Cp measurement using an automated biochemical analyzer. The severity of psychiatric symptoms was assessed using the Positive and Negative Syndrome Scale (PANSS), while the Tower of London (TOL) test was employed to evaluate executive function. Statistical analyses included one-way ANOVA and Spearman’s correlation to examine group differences and relationships between Cp levels and cognitive performance.

**Results:**

Significant differences in serum Cp levels were observed among the SC1, SC2, and control groups (P < 0.01). Executive function, assessed via the TOL test, showed no significant difference between the SC1 and SC2 groups (P > 0.05); however, both groups exhibited significantly impaired performance compared to the control group (P < 0.001). A strong positive correlation was identified between Cp levels and TOL performance in the SC1 group (r = 0.890, P < 0.001), particularly in simpler and moderately complex tasks.

**Conclusion:**

Based on the existing evidence of widespread cognitive impairment in schizophrenia patients, this study delves deeper into its potential causes. We found that regardless of whether schizophrenia patients had normal or low serum ceruloplasmin (CP) levels, their executive function was significantly lower than that of healthy individuals. Furthermore, a correlation analysis showed a significant link between CP levels and executive dysfunction in schizophrenia patients with decreased CP levels. Our study suggests that low CP levels may aggravate executive dysfunction, indicating that CP deficiency might be a biological marker of executive dysfunction in schizophrenia.

## 1 Introduction

Schizophrenia (SC) is a severe and chronic psychiatric disorder characterized by profound disturbances in cognition, thought processes, emotions, and behavior. Over the past century, schizophrenia has been conceptualized and defined in various ways across different cultures and psychiatric paradigms, reflecting its complexity and multifactorial nature. Currently, it is widely accepted that schizophrenia is a group of severe mental illnesses with unclear etiologies, marked by significant abnormalities in brain function and structure that lead to substantial occupational and social impairments, profoundly affecting patients’ quality of life and societal functioning (1, 2).

The clinical presentation of schizophrenia is highly heterogeneous and typically classified into three major symptom domains: positive symptoms (e.g., hallucinations and delusions), negative symptoms (e.g., social withdrawal and emotional blunting), and cognitive impairments. Among these, cognitive dysfunction has garnered increasing attention in recent years due to its pervasive nature and its critical impact on patients’ long-term prognosis and functional recovery (3). Cognitive function is a broad category that encompasses various processes of information processing by individuals, primarily including seven domains: basic cognitive processes such as information processing speed, attention/concentration, working memory, verbal learning and memory, visual learning and memory, reasoning and problem-solving, and social cognition. Executive function, however, represents a higher-order cognitive ability, mainly involving the regulation of complex behaviors such as planning, organization, decision-making, and inhibition. It is responsible for regulating and managing these basic cognitive processes to achieve individuals’ complex behaviors and goal-directed actions. Impairment of executive function is particularly detrimental to patients’ long-term prognosis and recovery of social function, thus attracting the attention of researchers (4).

The underlying neurobiological mechanisms contributing to cognitive deficits in schizophrenia are complex and not fully understood. Numerous studies have highlighted structural and functional abnormalities in brain regions such as the prefrontal cortex, hippocampus, and thalamus, which are known to play critical roles in cognitive functioning. Dysregulation of key neurotransmitter systems, including dopamine, glutamate, and gamma-aminobutyric acid (GABA), has also been implicated in the pathophysiology of cognitive dysfunction in schizophrenia (5, 6). Beyond neurotransmitter systems, cognitive function in schizophrenia patients correlates with brain-derived neurotrophic factor (BDNF) levels. Most studies indicate that schizophrenia is associated with cognitive impairment, and serum or plasma BDNF levels in these patients are lower than those in healthy control groups. This reduction in BDNF levels may be linked to neuronal damage, decreased synaptic plasticity, and neurotransmitter regulatory disorders, thereby contributing to impaired cognitive function (7–11). However, despite significant advances in understanding the neural underpinnings of cognitive deficits, specific biomarkers that reliably predict or correlate with cognitive impairments in schizophrenia remain elusive.

One promising area of research involves the role of serum ceruloplasmin (Cp) in cognitive dysfunction. Ceruloplasmin is a multi-copper oxidase synthesized primarily in the liver, where it plays a crucial role in maintaining copper and iron homeostasis. It regulates the transport and metabolism of these essential trace elements while protecting tissues from oxidative damage through its antioxidative properties (12). In addition to its systemic functions, Cp is involved in brain physiology, particularly in processes related to oxidative stress, neuroinflammation, and neuronal survival. Reduced serum Cp levels, or hypoceruloplasminemia, have been observed in several neurodegenerative disorders, including Wilson’s disease, Alzheimer’s disease, and Parkinson’s disease, where they are often associated with abnormal iron metabolism and excessive iron deposition in specific brain regions, leading to neuronal damage and cognitive decline (13, 14). A study published in Schizophrenia Research measured the plasma ceruloplasmin levels in 10 patients with schizophrenia and found them to be significantly higher than those in the healthy control group (15). A systematic review incorporating 59 articles compared serum trace element levels, including ceruloplasmin, between schizophrenia patients and healthy controls, revealing significantly different serum ceruloplasmin levels in schizophrenia patients compared to the healthy control group (16). These findings indicate that ceruloplasmin deficiency may exert its role in the pathophysiological process of schizophrenia through mechanisms such as inducing copper metabolic disorders, interfering with iron metabolism, increasing oxidative stress, affecting cellular signal transduction, and triggering neuroinflammation (17–19).

Despite the potential importance of Cp in schizophrenia, few studies have systematically investigated its relationship with cognitive dysfunction in this population. This study represents a novel effort to explore the association between reduced serum Cp levels and executive dysfunction in hospitalized schizophrenia patients. By utilizing the Tower of London (TOL) test, a widely recognized neuropsychological assessment tool, this study aims to provide a detailed characterization of the extent and nature of executive impairments in this population, while also examining the potential role of Cp as a biomarker for cognitive deficits. Specifically, this study seeks to: (1) compare serum Cp levels between schizophrenia patients and healthy controls; (2) evaluate differences in executive function across schizophrenia patients with varying Cp levels; and (3) assess the correlation between serum Cp levels and executive performance, particularly in tasks requiring problem-solving and planning.

Understanding the relationship between Cp and cognitive dysfunction in schizophrenia has significant clinical implications. If serum Cp is found to correlate with executive dysfunction, it could serve as a valuable biomarker for identifying patients at risk of severe cognitive impairments, facilitating early detection and intervention. Moreover, therapeutic strategies aimed at restoring or stabilizing Cp levels may represent a novel approach to mitigating cognitive decline and improving functional outcomes in schizophrenia patients. By integrating biochemical, neuropsychological, and clinical perspectives, this study seeks to advance the understanding of schizophrenia-related cognitive impairments and pave the way for innovative diagnostic and therapeutic strategies.

## 2 Materials and methods

### 2.1 Study population

This study recruited a total of 95 schizophrenia patients hospitalized at Shaoxing Seventh People’s Hospital between January 2023 and December 2024. These patients were divided into two groups based on their serum ceruloplasmin (Cp) concentrations: 48 patients with reduced serum Cp levels (designated as the SC1 group, Cp < 200 mg/L) and 47 patients with normal serum Cp levels (designated as the SC2 group, Cp 200-600 mg/L). Additionally, 42 age- and gender-matched healthy individuals were selected from the hospital’s physical examination center to serve as the control group.

#### 2.1.1 Inclusion criteria for the SC group

①. All participants met the diagnostic criteria for schizophrenia as defined by the fifth edition of the Diagnostic and Statistical Manual of Mental Disorders (DSM-5) and were confirmed using the Mini-International Neuropsychiatric Interview (MINI) version 6.0. ②. Patients were aged between 18 and 75 years. ③. Patients were capable of completing the Positive and Negative Syndrome Scale (PANSS) assessment and cognitive function tests, with a minimum PANSS score of 60.Informed consent was obtained from all participants, and the study was approved by the Ethics Committee of Shaoxing Seventh People’s Hospital. Exclusion Criteria for the SC Group: ①. Poor compliance or inability to complete assessments. ②. History of brain diseases, including cerebrovascular disorders or traumatic brain injury. ③. Severe systemic diseases affecting major organs (e.g., heart, liver, or kidneys). ④. Diagnosed Parkinson’s disease or Wilson’s disease.

#### 2.1.2 Inclusion criteria for the control group

①. Age and gender matched with the SC group. ②. No evidence of neurological disorders or focal signs during physical examination. ③. No history of psychiatric illnesses or other neurological diseases.

### 2.2 Assessment tools

#### 2.2.1 General data collection

Basic demographic and clinical information, including age, gender, and medical history, was collected for all participants. Diagnoses were confirmed independently by two experienced psychiatrists.

#### 2.2.2 Psychiatric symptom assessment

The PANSS was used to evaluate the severity of psychiatric symptoms in the SC group. This scale consists of positive, negative, and general psychopathology subscales, providing a comprehensive measure of symptom severity.

#### 2.2.3 Cognitive function assessment

Executive function was assessed using the Tower of London (TOL) test, a widely recognized neuropsychological tool designed to measure planning, problem-solving, and other executive functions. During the TOL test, participants were required to replicate specific ball arrangements shown in two images by determining the minimum number of moves necessary. Task difficulty was categorized into three levels: simple (1-2 moves), moderate (3-4 moves), and complex (5-6 moves). Participants’ performance was recorded based on accuracy and the number of correct answers across all difficulty levels.

### 2.3 Study design and quality control

All assessments were conducted in a quiet and comfortable room to minimize external disturbances. The purpose and procedures of the study were explained to participants before assessments began. Two trained psychiatrists, who underwent consistency training to minimize bias, administered all psychiatric and cognitive assessments. Each assessment took approximately 90 minutes to complete. Blood sample analyses were conducted by certified technicians using standardized protocols. All results were double-checked to ensure accuracy.

### 2.4 Laboratory measurements

Venous blood samples were collected from all participants after overnight fasting. Serum Cp levels were measured using an automated biochemical analyzer. All blood samples were processed within one hour of collection to ensure the accuracy of biochemical measurements.

### 2.5 Statistical analysis

Statistical analyses were performed using SPSS version 27.0. The following methods were used: 1. Group Comparisons: Serum Cp levels were compared among the SC1, SC2, and control groups using one-way analysis of variance (ANOVA).Differences in demographic variables (e.g., age and gender) were analyzed using chi-square tests and ANOVA, as appropriate. Comparisons of PANSS scores between the SC1 and SC2 groups were conducted using independent sample t-tests. 2. Correlation Analysis: The relationship between serum Cp levels and TOL test performance in the SC1 group was assessed using Spearman’s rank correlation coefficient. 3. Significance Thresholds:P-value of <0.05 was considered statistically significant. P-value of <0.001 indicated highly significant differences.

## 3 Results

### 3.1 General demographic information

The study included 95 hospitalized schizophrenia patients (SC group), consisting of 48 patients with reduced serum Cp levels (SC1 group) and 47 patients with normal serum Cp levels (SC2 group). Additionally, the control group consisted of 42 healthy individuals matched by age and gender. No significant differences were observed in age or gender distribution among the three groups (P > 0.05). The detailed demographic and clinical data are summarized in **Table 3**.

**Table 1:** TOL test performance across groups.

Group	n	Total score (mean ± SD)	Single-step	Two-steps	Three steps	Four steps	Five steps	Six steps
SC1	48	11.15 ± 2.60^a^	4.13 ± 0.61^a1^	3.02 ± 0.79^a2^	2.26 ± 0.79^a3^	1.64 ± 0.85^a4^	0.23 ± 1.46	0.00 ± 0.00
SC2	47	12.67 ± 3.71^b^	4.43 ± 0.63^b1^	3.38 ± 0.83^b2^	2.81 ± 0.99^b3^	1.86 ± 1.30^b4^	0.14 ± 0.36	0.05 ± 0.22
Control	42	15.73 ± 3.87^c^	4.68 ± 0.57^c1^	4.24 ± 0.58^c2^	3.46 ± 1.12^c3^	2.93 ± 1.44^c4^	0.32 ± 0.47	0.07 ± 0.26
F		20.288	9.309	30.636	14.427	13.917	0.356	1.659
P		<0.001	<0.001	<0.001	<0.001	<0.001	0.701	0.194

Post-hoc LSD test: a vs b (P=0.038), a vs c (P < 0.001), b vs c (P < 0.001).a1 vs b1(P=0.012), a1 vs c1 (P < 0.001), b1 vs c1 (P =0.058).a2 vs b2 (P=0.025),a2 vs c2 (P < 0.001), b2 vs C2 (P < 0.001).a3 vs b3 (P=0.03),a3 vs c3 (P < 0.001),b3 vs c3 (P=0.002).a4 vs b4 (P=0.394), a4 vs c4 (P < 0.001), b4 vs c4 (P < 0.001).

**Table 2:** Correlation analysis between Cp levels and TOL test scores in the 3 group.

Group	Correlation coefficient(r)						
	Total score	Single-step	Two steps	Three steps	Four steps	Five steps	Six steps
SC1	0.890***	0.764***	0.811***	0.689***	0.604***	0.126	0.00
SC2	0.420*	0.184	0.421*	0.446*	0.368	0.219	0.00
Control	0.055	0.008	0.079	0.09	0.163	0.177	0.210

* * * indicates P<0.001, * indicates P<0.05.

**Table 3:** General demographics and clinical characteristics.

Group	n	Age (mean ± SD)	Gender (M:F)	Disease duration (years, mean ± SD)
SC1 Group	48	45.85 ± 11.46	25:23	16.42 ± 7.83
SC2 Group	47	51.43 ± 12.13	23:24	19.74 ± 8.70
Control	42	50.51 ± 14.65	18:24	–
t/χ2	–	*F = 2.596*	*χ² = 0.780*	*t = -1.962*
P	–	*0.078*	*0.677*	*0.053*

The italicized numbers represent the statistical results.

### 3.2 Comparison of PANSS scores between SC1 group and SC2 group

The mean PANSS score of the SC1 group was 64.17 ± 2.98 points, and the mean score of the SC2 group was 64.77 ± 2.76 points. There was no statistically significant difference in scores between the SC1 and SC2 groups (P>0.05), indicating comparability. (See **Table 4**).

**Table 4:** PANSS scores for SC1 and SC2 groups.

Group	n	PANSS average value
SC1 Group	48	64.17 ± 2.98
SC2 Group	47	64.77 ± 2.76
t		-1.017
P		0.312

### 3.3 Serum ceruloplasmin levels

Significant differences in serum Cp levels were observed among the SC1 group, SC2 group, and control group (P < 0.01). *Post-hoc* analyses revealed that Cp levels in the SC1 group were significantly lower than those in the SC2 group and the control group (P < 0.01), there is also a significant difference in serum ceruloplasmin between the full schizophrenia group (SC1+SC2) and healthy controls (P < 0.01), while no significant difference was found between the SC2 group and the control group. Detailed results are shown in **Table 5**.

**Table 5:** Comparison of serum Cp levels among groups.

Group	n	Serum Cp Levels (mg/L, mean ± SD)
SC1 Group	48	175.24 ± 27.59^a^
SC2 Group	47	243.55 ± 38.76^b^
Control	42	267.23 ± 38.29^c^
F		*84.504*
P		0.000

Post-hoc LSD test: a vs b (P < 0.01), a vs c (P < 0.01), b vs c (P > 0.05), a+b vs c(P < 0.01).

### 3.4 Executive function performance

Performance on the Tower of London (TOL) test was used to assess executive function. No significant difference was observed in overall TOL scores between the SC1 and SC2 groups (P > 0.05); however, both groups performed significantly worse than the control group (P < 0.001). Further analysis of task difficulty levels revealed significant differences between the SC1 and SC2 groups for simple (1-2 moves) and moderate (3-4 moves) tasks (P < 0.05). Detailed results are presented in **Table 1**.

### 3.5 Correlation between serum Cp levels and executive function

A strong positive correlation was observed between serum Cp levels and TOL scores in the SC1 group (r = 0.890, P < 0.001). This correlation was particularly strong for simpler and moderately complex tasks (P < 0.001), while no significant correlation was observed for complex tasks (P > 0.05). Detailed correlation results are shown in **Table 2**.

## 4 Discussion

This study provides compelling evidence for the association between reduced serum ceruloplasmin (Cp) levels and executive dysfunction in hospitalized schizophrenia patients. The findings underscore the potential role of Cp as a biomarker for cognitive impairments in this population, offering new insights into the biochemical underpinnings of schizophrenia-related cognitive deficits.

Cognitive dysfunction, particularly executive dysfunction, is widely recognized as a core feature of schizophrenia, affecting patients’ ability to plan, problem-solve, and adapt to changing circumstances. The results of this study are consistent with previous research demonstrating significant impairments in executive functioning among schizophrenia patients compared to healthy controls (4, 20, 21). Using the Tower of London (TOL) test, this study revealed that schizophrenia patients, regardless of serum Cp levels, performed significantly worse than healthy individuals in tasks requiring planning and problem-solving. These deficits were particularly pronounced in simpler and moderately complex tasks, suggesting that schizophrenia patients may struggle more with cognitive efficiency rather than task complexity. This finding aligns with previous studies emphasizing the role of the prefrontal cortex in executive dysfunction in schizophrenia (22, 23).

One of the most significant findings of this study is the strong positive correlation between serum Cp levels and TOL performance in the SC1 group. Patients with reduced serum Cp levels exhibited poorer executive function compared to those with normal Cp levels, suggesting that Cp depletion exacerbates cognitive deficits in schizophrenia. Ceruloplasmin is a multi-functional protein involved in copper and iron homeostasis, as well as oxidative stress regulation. Hypoceruloplasminemia (reduced Cp levels) has been implicated in various neurological disorders, including Wilson’s disease, Alzheimer’s disease, and Parkinson’s disease, where it is associated with abnormal iron metabolism and subsequent neuronal damage (12–14). The results of this study extend these findings to schizophrenia, highlighting Cp’s potential role in the pathophysiology of cognitive impairments.

The link between reduced Cp levels and executive dysfunction may be explained by several mechanisms.

First, Cp is critical for maintaining iron homeostasis by facilitating the oxidation of ferrous iron (Fe2+) to ferric iron (Fe3+), thereby preventing iron accumulation and oxidative damage in neural tissues. Reduced Cp levels can disrupt this balance, leading to iron deposition in brain regions such as the prefrontal cortex, which plays a central role in executive functioning. Iron deposition has been associated with increased oxidative stress, mitochondrial dysfunction, and neuronal apoptosis, all of which contribute to cognitive impairments (24, 25).

Second, Cp also plays a role in regulating the copper-zinc balance, which is essential for synaptic plasticity and neurotransmitter release. Disruptions in this balance may impair synaptic signaling and neuronal communication, further exacerbating cognitive deficits. Previous studies have shown that both copper and zinc deficiencies are associated with cognitive impairments, including memory loss and executive dysfunction, providing additional support for the role of Cp in maintaining cognitive health (26, 27).

The findings of this study have several important clinical implications.

First, serum Cp levels is expected to serve as a potential biomarker for identifying schizophrenia patients at risk of severe cognitive impairments. Our proposal of serum ceruloplasmin as a potential biological marker is mainly based on the observed correlation between ceruloplasmin levels and cognitive impairments, especially in the early stages of cognitive decline. We don’t advocate replacing routine cognitive assessments with ceruloplasmin measurement. Instead, we emphasize its possible value in assisting in the identification of high-risk patients, particularly in clinical settings with limited resources or where comprehensive neuropsychological testing isn’t feasible.

Second, Routine monitoring of Cp levels may enable clinicians to implement timely interventions aimed at mitigating cognitive decline and improving functional outcomes. Multiple studies have found that ceruloplasmin is correlated with cognitive decline in neurodegenerative diseases such as Alzheimer’s disease (28–32). A prospective study has suggested that elevated serum ceruloplasmin levels in neurodegenerative diseases may precede the onset of cognitive symptoms, offering a potential biomarker for early prediction of cognitive dysfunction (33).

In addition, therapeutic strategies targeting Cp restoration or stabilization could represent a novel approach to enhancing cognitive function in schizophrenia patients. For example, interventions such as antioxidant therapy or trace metal supplementation may help restore Cp levels and mitigate oxidative stress, thereby improving cognitive outcomes (34, 35).

Moreover, the TOL test proved to be an effective tool for assessing executive dysfunction in schizophrenia patients. Its ability to differentiate between patients with varying degrees of cognitive impairment suggests its potential utility in clinical and research settings for evaluating treatment efficacy and monitoring disease progression.

## 5 Limitations and future directions

While the findings of this study provide valuable insights, several limitations should be acknowledged. First, the sample size was relatively small, which may limit the generalizability of the results. Future studies with larger, more diverse cohorts are needed to validate these findings. Second, this study focused exclusively on hospitalized patients, who may represent a more severe subset of the schizophrenia population. Including outpatients or individuals in the early stages of the disease could provide a more comprehensive understanding of the relationship between Cp levels and cognitive impairments.

Additionally, this study employed a single neuropsychological tool (TOL test) to assess executive function. Although the TOL test is widely recognized for its sensitivity to executive dysfunction, incorporating additional cognitive assessments (e.g., Wisconsin Card Sorting Test, Stroop Test) could provide a more nuanced understanding of cognitive deficits in schizophrenia. Furthermore, integrating neuroimaging techniques, such as magnetic resonance imaging (MRI) or positron emission tomography (PET), could help elucidate the structural and functional brain changes associated with reduced Cp levels.

Finally, the cross-sectional design of this study limits its ability to establish causal relationships between Cp levels and executive dysfunction. Longitudinal studies are needed to determine whether changes in Cp levels over time are associated with cognitive decline or improvement in schizophrenia patients.

This study provides evidence supporting the correlation between reduced serum ceruloplasmin (Cp) levels and executive dysfunction in schizophrenia patients. Patients with lower Cp levels demonstrated significantly poorer performance on executive function tasks, particularly in simpler and moderately complex problem-solving scenarios. These findings suggest that Cp depletion may contribute to cognitive deficits in schizophrenia by disrupting metal homeostasis, increasing oxidative stress, and impairing neuronal signaling.

The results highlight the potential of serum Cp as a biomarker for identifying schizophrenia patients at risk of severe cognitive impairments. Regular monitoring of Cp levels in clinical practice could help guide personalized treatment strategies aimed at improving cognitive and functional outcomes. Furthermore, therapeutic interventions targeting Cp restoration, such as antioxidant therapy or trace metal supplementation, warrant further investigation as potential approaches to mitigating executive dysfunction in schizophrenia.

Future research should focus on expanding sample sizes, including broader patient populations, and employing longitudinal designs to validate and extend these findings. Integrating biochemical analyses with advanced neuroimaging techniques could also provide deeper insights into the structural and functional brain changes associated with Cp depletion. By advancing our understanding of the role of Cp in schizophrenia-related cognitive impairments, these efforts have the potential to inform novel diagnostic and therapeutic strategies.

## Data Availability

The raw data supporting the conclusions of this article will be made available by the authors, without undue reservation.
